# New Properties and Mitochondrial Targets of Polyphenol Agrimoniin as a Natural Anticancer and Preventive Agent

**DOI:** 10.3390/pharmaceutics13122089

**Published:** 2021-12-05

**Authors:** Tatiana A. Fedotcheva, Olga P. Sheichenko, Nadezhda I. Fedotcheva

**Affiliations:** 1 Science Research Laboratory of Molecular Pharmacology, Medical Biological Faculty, Pirogov Russian National Research Medical University, Ministry of Health of the Russian Federation, Ostrovityanova St. 1, Moscow 117997, Russia; tfedotcheva@mail.ru; 2 All-Russian Research Institute of Medicinal and Aromatic Plants, Gryna St. 7, Moscow 117216, Russia; sheychenko@vilarnii.ru; 3 Institute of Theoretical and Experimental Biophysics, Russian Academy of Sciences, Institutskaya Str. 3, Pushchino142290, Russia

**Keywords:** agrimoniin, mitochondria, cancer cell, swelling, respiration, mitochondrial permeability transition pore

## Abstract

Agrimoniin is a polyphenol from the group of tannins with antioxidant and anticancer activities. It is assumed that the anticancer action of agrimoniin is associated with the activation of mitochondria-dependent apoptosis, but its mitochondrial targets have not been estimated. We examined the direct influence of agrimoniin on different mitochondrial functions, including the induction of the mitochondrial permeability transition pore (MPTP) as the primary mechanism of mitochondria-dependent apoptosis. Agrimoniin was isolated from *Agrimonia pilosa* Ledeb by multistep purification. The content of agrimoniin in the resulting substance reached 80%, as determined by NMR spectroscopy. The cytotoxic effect of purified agrimoniin was confirmed on the cultures of K562 and HeLa cancer cells by the MTT assay. When tested on isolated rat liver mitochondria, agrimoniin at a low concentration (10 µM) induced the low-amplitude swelling, which was inhibited by the MPTP inhibitors ADP and cyclosporine A, activated the opening of MPTP by calcium ions and stimulated the respiration supported by succinate oxidation. Also, agrimoniin reduced the electron acceptor DCPIP in a concentration-dependent manner and chelated iron ions. Owing to all these properties, agrimoniin can stimulate apoptosis or activate mitochondrial functions, which can be helpful in the prevention and elimination of stagnant pathological states.

## 1. Introduction

Agrimoniin is a polyphenol from the group of hydrolyzable tannins. Agrimoniin differs from other tannins in its high molecular weight and the largest number of hydroxyl groups in the molecule. It is known that the compounds of this group have high biological activity, including antioxidant, anti-inflammatory, antitumor, and others [1,2].

The antioxidant properties of tannins have been extensively researched. It was found that the antioxidant activity of these compounds is associated with the inhibition of pro-oxidant enzymes, such as lipoxygenase, xanthine oxidase, and monoamine oxidase, with the chelation of transition metals, suppression of lipid peroxidation, and the binding of hydroxyl radicals [1,2,3]. Recent investigations aimed at studying the specific mechanisms and targets of tannins’ action found that the effect of these compounds is associated not only with antioxidant activity but also with other properties manifested in the regulation of signaling pathways, proliferation, gene expression, and metabolism. These effects are believed to be mediated primarily by direct binding to specific proteins and enzymes. The binding of tannins to phosphatase [4], pyruvate kinase [5], gluten proteins [6], albumin [7], and deacetylase [8] was revealed. It was shown that the binding of tannins to kinases and phosphatases led to changes in the level of kinase phosphorylation and, accordingly, the activity of signaling pathways involved in the regulation of proliferation and apoptosis [9]. Direct binding of tannin to the lysine residue of pyruvate kinase blocked the metabolic activity of the enzyme [5]. The influence of tannins on the expression of genes involved in the regulation of metabolism was also shown, manifesting itself in the modulation of the activity of deacetylases and a decrease in the expression of the enzymes of glycolysis and lipogenesis [10,11]. Furthermore, tannins reduced inflammation by decreasing the expression of proinflammatory mediators and cytokines [12] and suppressing the biosynthesis of prostaglandins [13].

Moreover, tannins exhibit anticancer activity, affecting apoptosis, proliferation, invasion, and metastasis [14]. Recent studies have demonstrated that mitochondria are involved in the cytotoxic action of tannins [15,16]. The mitochondria-associated cytotoxic effect of these compounds has been observed in a number of tumor lines. It has been shown that tannins, including agrimoniin, activate mitochondria-dependent apoptosis and cause disturbances in intracellular calcium homeostasis, mitochondrial membrane potential, and energy production [17,18,19]. As seen in some tumor lines, this activation of apoptosis is accompanied by a significant increase in the expression of proapoptotic proteins and caspases and a decrease in the expression of antiapoptotic factors [19,20]. With regard to the direct action of tannins on isolated mitochondria, the inhibitory effect of some of them on respiration, oxidative phosphorylation, and lipid peroxidation was previously revealed [21,22]. Changes observed in the respiration activity depended on the oxidation substrate and varied from activation to inhibition, depending on the polyphenol concentration and the state of mitochondria.

Previously, the anticancer and immunomodulatory effects of agrimoniin isolated from the roots of *Agrimonia pilosa* Ledeb (Rosaceae) were reported [23,24,25]. Aqueous extracts of the aerial parts of *A. pilosa* have shown antiviral activity against hepatitis B viruses [26], as well as anticoagulant activity [27]. Water-alcoholic extracts of *A. pilosa* leaves demonstrated antioxidant and anti-inflammatory activities [28]. It was found that agrimoniin (C_82_H_54_O_52_), with a molecular weight of 1871.282, is a dimeric ellagitannin responsible for the antitumor activity of *A. pilosa* Ledeb [17,28]. The substance was low in toxicity, and with LD50 equal to 100 mg/kg and over1000 mg/kg, it was administered to mice peritoneal and orally, respectively [28].

Although the anticancer action of agrimoniin is apparently associated with the activation of mitochondria-dependent apoptosis, its influence on isolated mitochondria has not been previously studied. In this work, we examined the effect of agrimoniin on respiration, the activity of succinate dehydrogenase, a key enzyme of the tricarboxylic acid cycle, and the induction of the Ca-dependent cyclosporine-sensitive mitochondrial pore as the primary mechanism of mitochondria-dependent apoptosis. A study of the cytotoxic effect of agrimoniin was carried out on the cultures of K562 and HeLa tumor cells. The chelating properties of agrimoniin were also studied. New properties and mitochondrial targets of agrimoniin, as an antitumor agent, were revealed.

## 2. Materials and Methods

Preparation and Characterization of the Quality of Agrimoniin. Agrimoniin was isolated from A. pilosa Ledeb. A perennial herb of the Rosaceae family has been widely used in medical practice in China, Japan, and Korea. A. pilosa Ledeb was cultivated on the territory of the botanical garden at the All-Russian Scientific Research Institute of Medicinal and Aromatic Plants. Hydrolyzable galloellagotannin agrimoniin was obtained from the aerial part of the hairy agrimony and harvested in the flowering phase (the beginning of fruiting), according to the method described [29]. The extraction was carried out in several stages, including water–ethanol, n-butanol-petroleum ether, and water–acetone mixtures. Resinous precipitates were removed from the technological process at each stage of extraction. The aqueous–acetone solution was filtered out, evaporated, dissolved in distilled water, and treated first with ethyl acetate three times and then with n-butanol. The aqueous phase, after treatment with solvents, was evaporated. Thin-layer chromatography was carried out on cellulose F (1.05565, Merck, Darmstadt, Germany) in a solvent system of water and glacial acetic acid (95:5 by volume). The detection of agrimoniin was carried out with a solution of 1% ferric chloride in ethyl alcohol. The fractions giving blue color with iron were selected and purified by column chromatography on Filtrack cellulose in a ratio of 1:40 with the elution by water. The fraction giving blue color with iron was identified on TLC as a single spot. Final purification included ion-exchange chromatography. The content of agrimoniin in the samples was determined by the NMR method.

**Figure 1:**
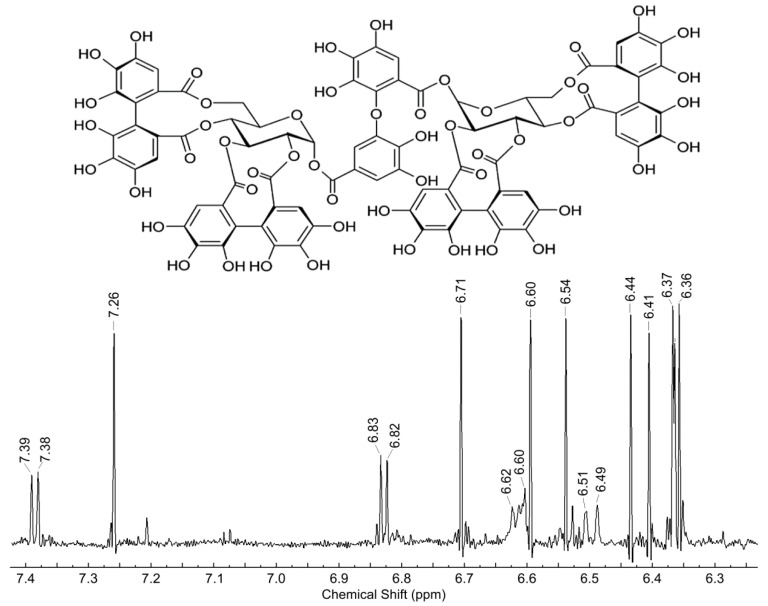
Structure and fragment of 1H NMR spectrum (200 MHz, CD3OD) of aromatic and two H-1 glucose protons of extracted agrimoniin.

Agrimoniin consists of two pedunculagin residues attached to carbon atoms at C-1 of glucose molecules through dehydrodigallic acid (Figure 1). Figure 1 shows a fragment of the 1H NMR spectrum (200 MHz, CD3OD) of aromatic and two H-1 protons of the resulting compound for the purpose of its identification. The specificity of the spectrum was determined by comparing the values of chemical shifts and the interaction constants of signals of eleven aromatic and two H-1 glucose protons of the resulting agrimoniin with the corresponding literature data [29]. The content of agrimoniin in the substance was determined by the NMR method. Fellamurin (basic substance content 99.0%) was used as an internal standard. The values of the integrals of agrimoniin protons were compared at 7.26 ppm (singlet, 1H) and protons of the methyl group of the standard (1.59 ppm, singlet, 3H). The calculations were carried out as described in the State Pharmacopoeia of the Russian Federation. XIV edition [https://pharmacopoeia.ru/ofs-1-2-1-1-0007-15-spektroskopiya-yadernogo-magnitnogo-rezonansa/] (accessed on 20 November 2021). According to calculations, the content of agrimoniin in the resulting substance reached 80%. The dominant impurities are sugars (range 3.2–4.2 ppm).

Preparation of Rat Liver Mitochondria. Mitochondria were isolated from adult Wistar male rats. The study was conducted following the ethical principles formulated in the Helsinki Declaration on the care and use of laboratory animals. Manipulations were carried out by the certified staff of the Animal Department of the Institute of Theoretical and Experimental Biophysics (Russian Academy of Sciences and approved by the Commission on Biomedical Ethics of ITEB RAS (N28/2021, 9 January 2021). During the study, the animals were kept in wiremesh cages at room temperature (22 °C) with a light/dark cycle of 12 h. Mitochondria from the liver of anesthetized animals were isolated using the standard method [14]. The liver was rapidly removed and homogenized in an ice-cold isolation buffer containing 300 mM sucrose, 1 mM EGTA, and 10 mM HEPES–Tris (pH 7.4). The homogenate was centrifuged at 600× *g* for 7 min at 4 °C, and the supernatant fraction was then centrifuged at 9000× *g* for 10 min to obtain mitochondria. Mitochondria were washed twice in the above medium without EGTA and BSA. The final mitochondrial pellet was suspended in the washing medium to yield 60 mg protein/mL and kept on ice for analysis.

Determination of Respiration Rates. Oxygen consumption in a mitochondrial suspension was determined by the polarographic method with a Clark-type electrode in a closed chamber of 1 mL containing 1.0–1.2 mg of mitochondrial protein under continuous stirring. Mitochondrial respiration was supported by succinate (5 mM) or glutamate (5 mM) plus malate (1 mM) and activated by ADP (200 µM) and DNP (50 µM) for the evaluation of phosphorylating and uncoupled respiration, respectively. The respiratory control index was defined as the ratio of the ADP-stimulated respiration rate to the respiration rate after ADP phosphorylation.

Determination of SDH Activity. The activity of SDH was determined by the reduction of the electron acceptor DCPIP [30]. Mitochondria (0.5 mg protein/mL) were incubated in 2 mL of medium containing 125 mM KCl, 15 mM HEPES, pH 7.4 in the presence of 1 mM cyanide, 20 µL of 10% Triton X-100, and 100 µM DCPIP. The DCPIP reduction was induced by adding 5 mM succinic acid with further activation by 100 µM PMS. The acceptor reduction rate was measured at a wavelength of 600 nm using an Ocean Optics USB4000 spectrophotometer.

Determination of the Ca^2+^-induced MPTP Opening. MPTP opening was induced by the sequential loading of the incubation medium with CaCl_2_ [31]. The opening of the MPTP was registered as a steep rise in calcium ions in the incubation medium and a decline of the mitochondrial membrane potential. The difference in the electric potential on the inner mitochondrial membrane was measured from the redistribution of lipophilic cation tetraphenylphosphonium (TPP+) between the incubation medium and mitochondria. The concentration of TPP+ in the incubation medium was recorded by a TPP+ selective electrode (Nico, Moscow, Russia). Changes in the calcium ion concentration in the incubation medium were recorded by a Ca^2+^-selective electrode (Nico, Moscow, Russia). The concentration of TTP+ and Ca^2+^ was registered simultaneously in an open chamber of volume 1 mL containing 1.0–1.2 mg of mitochondrial protein under continuous stirring. The mitochondrial calcium retention capacity (CRC) was defined as the total concentration of added Ca^2+^ required for pore opening. The incubation medium contained 125 mM KCl, 15 mM HEPES, 1.5 mM phosphate, 5 mM succinate, and pH 7.25.

Determination of Mitochondrial Swelling. The swelling of mitochondria was measured at a wavelength of 540 nm using an Ocean Optics USB4000 spectrophotometer. The swelling was assessed by measuring the changes in optical density during incubation [32]. Mitochondria at a mitochondrial protein concentration of 0.3–0.4 mg/mL were incubated in the same buffer (125 mM KCl, 15 mM HEPES, 1.5 mM phosphate, and 5 mM succinate).

Cell Viability Assay. HeLa and K562 cell lines were used to test agrimoniin cytotoxicity. HeLa and K562 lines are appropriate cell lines usually used in the first stage of anticancer drugs screening [33], while the cell culture of normal skin fibroblasts is used as an object for testing agrimoniin cytotoxicity on noncancer cells [34]. All cell lines were obtained from All-Russian Research Institute of Medicinal and Aromatic Plants biocollection bank, http://vilarnii.ru/biokollektsii/ (accessed on 20 November 2021). Briefly, neonatal Wistar rats (7-dayold male) were used to obtain the cells from the skin of fetuses from the back. The skin fragments in aseptic conditions were placed in a 0.25% trypsin solution, incubated for 10 min at 37 °C, then transferred into DMEM containing 20% fetal calf serum (Research Grade, Auckland, New Zealand), L-glutamine at a concentration of 100 μg/mL and antibiotics gentamicin sulfate (Dalhimpharm, Khabarovsk, Russia) and streptomycin sulfate (Biochimic, Saransk, Russia) at a concentration of 40 μg/mL and disaggregated into single cells using vibration. The resulting cell suspension was washed from trypsin by centrifugation (5 min at 150× *g*). The cell pellet was suspended in DMEM containing 20% fetal calf serum, antibiotics gentamicin sulfate, and streptomycin sulfate (40 μg/mL each). The obtained fibroblasts after the third passage were used in the experiments.

The cytotoxicity of agrimoniin was estimated using the MTT assay. Cells were incubated at 37 °C under 5% CO_2_, using standard DMEM medium (Merck, Darmstadt, Germany) supplemented with 10% thermally inactivated fetal calf serum (Research Grade, Auckland, New Zealand), l-glutamine at a concentration of 100 μg/mL and antibiotics gentamicin sulfate (Dalhimpharm, Khabarovsk, Russia), and streptomycin sulfate (Biochimic, Saransk, Russia) at a concentration of 40 μg/mL. Agrimoniin was dissolved in a DMEM medium. Solutions of agrimoniin in DMEM medium were added to final concentrations of 10^−9^–10^−3^ M and incubated for 48 and 72 h with the cells in 96-well plates. At the end of incubation, the viability of the culture was assessed using the standard MTT test [35]. Color development was determined by measuring the optical density at a wavelength of 530 nm on a microplate reader (enzyme-linked immunosorbent assay “UNIPLAN” AIFR-01 (Pikon, Moscow, Russia). The IC_50_ values were obtained by linear regression analysis using the program GraphPad Prism (version 9, Graphpad Software Inc, San Diego, CA, USA).

Statistical Analysis. The data given represent the means ± standard error of means (SEM) from five to seven experiments or are the typical traces of three to five identical experiments using different mitochondrial preparations. The statistical significance was estimated by the Student’s *t*-test with *p* < 0.05 as the criterion of significance.

## 3. Results

### 3.1. Influence of Agrimoniin on Mitochondrial Respiration and SDH Activity

**Figure 2:**
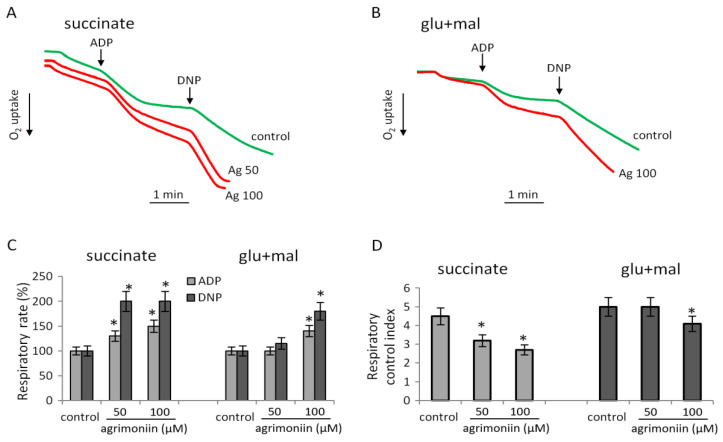
Influence of agrimoniin on respiration during oxidation of succinate and NAD-dependent substrates. Respiration supported by succinate (5 mM) oxidation (**A**) and glutamate (4 mM) plus malate (2 mM) oxidation (**B**) in control and in the presence of agrimoniin (50–100 µM); effect of agrimoniin in different concentrations on respiration rates in ADP (200) and DNP (50 µM) –stimulated states (**C**); respiratory control index (**D**). The respiratory control index was calculated as the ratio of respiration in the course and after ADP phosphorylation. Asterisk (*) indicates values that differ significantly from the control values (*p* < 0.05).

Respiratory rates in ADP-stimulated and disconnected states, and respiratory control, were measured during the oxidation of succinate, a substrate of II complex, and NAD-dependent substrates glutamate with malate, substrates of the complex I of the respiratory chain. Figure 2A shows the original curves of oxygen uptake during the oxidation of succinate in the control and after the addition of agrimoniin at concentrations of 50 μM and 100 μM. At these concentrations, agrimoniin activated ADP-stimulated respiration by 40% and 50%. To an even greater extent, almost twofold, agrimoniin activated DNP-stimulated respiration, and the respiration rate reached its maximum values already at 50 μM. The respiration activation on succinate significantly exceeded the activation during the oxidation of glutamate with malate, which was observed at higher concentrations of agrimoniin (Figure 2B). The rate of ADP-stimulated respiration increased by 30% and that of DNP-stimulated respiration by 100% upon oxidation of succinate in the presence of 50 μM of agrimoniin, while for the activation of respiration during oxidation of NAD-dependent substrates, 100 μM agrimoniin was required (Figure 2C). Respiratory control, which reflects the efficiency of oxidative phosphorylation, decreased on both types of substrates in the same ratio (Figure 2D). These data show that agrimoniin has an activating effect on mitochondrial respiration, mainly on the second complex of the respiratory chain associated with the oxidation of succinate. The subsequent experiments attempted to determine to what extent this effect can be associated with the influence of agrimoniin on the activity of SDH.

**Figure 3:**
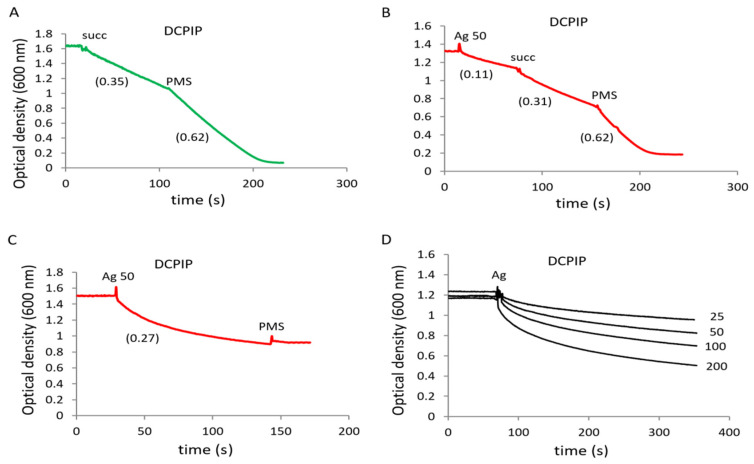
Influence of agrimoniin on succinate dehydrogenase activity. SDH activity in rat liver mitochondria (1 mg protein/mL) was determined by DCPIP reduction in control (**A**) and in the presence of 50 µM agrimoniin (**B**). DCPIP reduction was induced by succinate (5 mM) addition and was activated by PMS (100 µM) addition. DCPIP reduction by 50 µM agrimoniin (**C**) and in the range of concentrations 25–200 µM (**D**) in buffer medium without mitochondria.

The SDH activity was measured by reducing the electron acceptor DCPIP after successive additions of succinate and the intermediate electron carrier PMS. The acceptor reduction occurred in response to succinate addition and was accelerated with the addition of PMS (Figure 3A). In the presence of agrimoniin, DCPIP reduction was observed in the absence of the substrate and was activated after the addition of succinate and PMS (Figure 3B).

The results show that agrimoniin can reduce DCPIP not only in the absence of a substrate but even in the absence of mitochondria, wherein PMS did not cause an additional reduction of the acceptor (Figure 3C). These data indicate that agrimoniin directly reduces DCPIP by acting as an electron donor for this acceptor. Moreover, the degree of DCPIP reduction depended on the concentration of agrimoniin and rose from 24% to 70%, with an increase in its concentration from 25 to 200 µM (Figure 3D).

### 3.2. Influence of Agrimoniin on Calcium-Induced MPTP Opening

**Figure 4:**
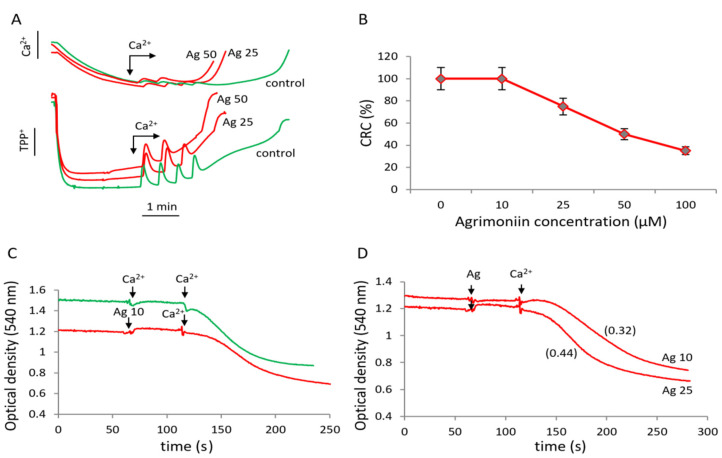
Influence of agrimoniin on MPTP opening induced by calcium ions. (**A**)influence of agrimoniin (Ag) on membrane potential (lower curves) and induction of calcium efflux (upper curves) in the course successive CaCl_2_ additions of 25 µM each in the control (green curves) and after agrimoniin (25 µM and 50 µM) additions (red curves). (**B**)calcium retention capacity (CRC)decreased by agrimoniin at different concentrations; the data represent the average values from five experiments. (**C**) induction of mitochondrial swelling by calcium ions in the control (green curve) and in the presence of 10 µM agrimoniin (red curve). (**D**) swelling rates in the presence of 10 µM and 25 µM agrimoniin. Swelling rates are indicated in parentheses.

MPTP induction is one of the main factors in mitochondria-dependent cell death. In this regard, the study of the effect of agrimoniin on MPTP opening is of great importance for elucidating the role of mitochondria in apoptosis induced by agrimoniin. Pore opening was assessed by a decrease in the membrane potential, the release of calcium ions, and the swelling of mitochondria in response to successive calcium supplementation in control samples and in the presence of agrimoniin in the concentration range of 10–100 μM (Figure 4).

Agrimoniin decreased the threshold concentration of calcium ions required for pore opening, causing a drop in the membrane potential and the release of calcium ions at lower calcium concentrations compared with control (Figure 4A). The effect was highly dependent on the concentration of agrimoniin (Figure 4B). The ability of mitochondria to accumulate calcium ions was decreased by 25% and 50% at agrimoniin concentrations of 25 and 50 μM, respectively.

A similar effect was observed in experiments with the mitochondrial swelling stimulated by calcium ions. The swelling in control samples was induced by adding 50 μM calcium, in the presence of 10 μM agrimoniin, which occurred already at a calcium concentration of 25 μM (Figure 4C). At this calcium ions concentration, the swelling rate increased by 50%, increasing agrimoniin concentration from 10 to 25 μM (Figure 4D). Thus, low concentrations of agrimoniin stimulate the calcium ion-induced swelling of mitochondria and MPTP opening.

### 3.3. Effect of Agrimoniin on the Mitochondrial Swelling

**Figure 5:**
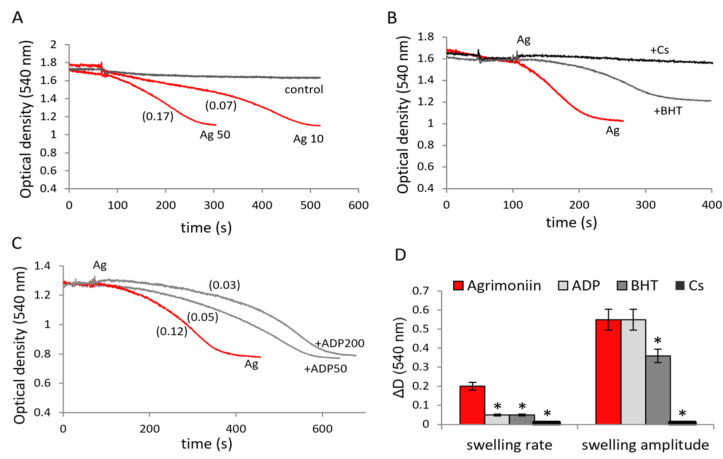
Induction of the mitochondrial swelling by agrimoniin and the influence of MPTP inhibitors. Induction of the swelling of mitochondria by additions of 10 µM and 50 µM agrimoniin (**A**); the influence of butyl-hydroxytoluene (BHT, 10 µM), cyclosporine A (Cs, 2 µM) (**B**) and ADP (50 and 200 µM) (**C**) on the induction of swelling by agrimoniin; D influence of inhibitors on the rate and amplitude of the swelling induced by agrimoniin (**D**). Swelling rates (ΔD/min) are indicated in parentheses. Asterisk (*) indicates values that differ significantly from the control values (*p* < 0.05).

Considering that, even at low concentrations, agrimoniin activates calcium-induced swelling in the following experiments, we tested the effect of agrimoniin on the swelling in the absence of calcium ions. Indeed, agrimoniin itself induced the swelling, which was characterized by a significant lag phase and a low rate but strongly depended on the concentration of agrimoniin (Figure 5A). The lag phase disappeared, and the swelling rate increased 2.5 times with an increase in the concentration of agrimoniin from 10 to 50 μM. This high-amplitude swelling was completely inhibited in the presence of cyclosporin A and partially in the presence of BHT, a lipid radical scavenger (Figure 5B). As can be seen, the BHT effect manifested not only in a sharp decrease in the swelling rate but also in a twofold decrease in the swelling amplitude. In addition, agrimoniin-induced swelling was effectively inhibited by ADP, another known MPTP inhibitor (Figure 5C). ADP reduced the swelling rate 3-fold and 6-fold at concentrations of 50 and 200 μM, respectively, although the swelling amplitude after a prolonged incubation reached the value observed in the absence of inhibitors. Thus, according to the degree of inhibition of agrimoniin-induced swelling, and considering its rate and amplitude, the inhibitors are ranked as follows in order of decreasing effectiveness: Cs > BHT > ADP (Figure 5D).

### 3.4. Effect of Agrimoniin on the Mitochondrial Swelling in the Presence of Iron Ions

**Figure 6:**
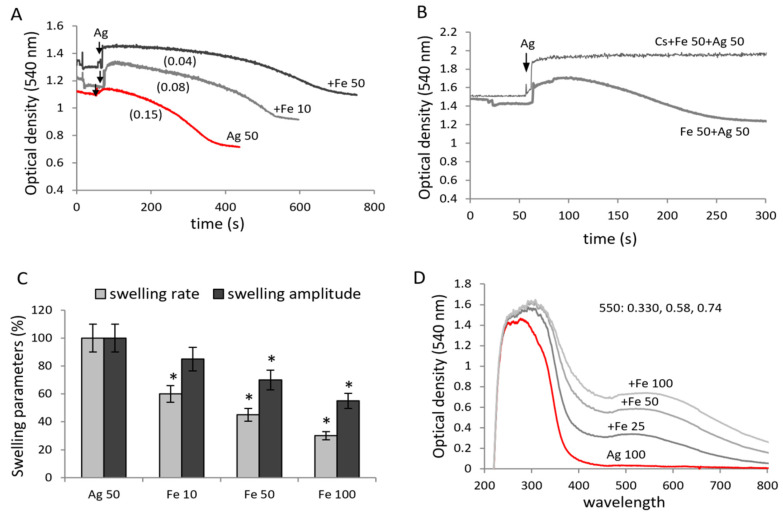
Influence of agrimoniin on the mitochondrial swelling in the presence of iron ions. Swelling of mitochondria induced by agrimoniin (50 µM) in the presence of 10 µM and 50 µM FeSO_4_ (**A**); inhibition of swelling induced by agrimoniin + iron (**B**); swelling rates (ΔD/min) and swelling amplitude (ΔD) during the combined additions of 50 µM agrimoniin and iron ions at different concentrations (**C**); changes in spectral characteristics of agrimoniin after addition of iron (**D**). Swelling rates (ΔD/min) are indicated in parentheses. Asterisk (*) indicates values that differ significantly from the control values (*p* < 0.05).

An important known property of polyphenols is their interaction with iron ions. This aspect has not been investigated for agrimoniin. Since agrimoniin, as shown above, induces pore opening, and iron ions, according to different data, sensitize MPTP opening [36,37], it could be expected that the effect in the presence of both factors will increase. In contrast, the addition of iron (FeSO_4_) caused the inhibition of agrimoniin-induced swelling (Figure 6A). At an agrimoniin concentration of 50 μM, the swelling rate decreased two and six times at iron concentrations of 10 and 50 μM, respectively. Although increasing iron concentration decreased both the rate and amplitude of agrimoniin-induced swelling, complete inhibition was not observed (Figure 6B). Swelling in the presence of both factors was entirely inhibited by cyclosporine A (Figure 6C). It is important to note that the addition of iron in the presence of agrimoniin caused an increase in optical density associated with the appearance of blue color in all samples, which was also observed visually. The appearance of a blue color, according to the literature data, is associated with the formation of iron–polyphenol complexes accompanied by the appearance of an absorption peak in the region of 550–570 nm. A similar effect of the interaction of agrimoniin with iron ions is also observed in spectral measurements. The addition of iron to agrimoniin was also accompanied by the appearance of blue color and an increase in absorption at 455 nm (Figure 6D). The formation of these complexes likely decreases the influence of every single component on their mitochondrial targets.

### 3.5. Cytotoxic Effect of Agrimoniin on Cancer Cell Lines

In order to compare the effectiveness of the preparation toward mitochondria and tumor cells, we tested its possible cytotoxic activity on K562 and HeLa cell cultures. Rat skin fibroblasts served as a control. The cells were incubated with agrimoniin in the range of concentrations from 1 × 10^−10^ to 1 × 10^−3^ M.

**Figure 7:**
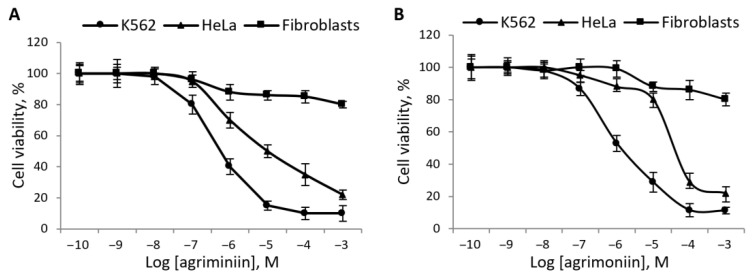
Influence of agrimoniin on the viability of cancer cells and fibroblasts. Cells of cancer lines HeLa and K562, and rat skin fibroblasts were incubated during 48 h (**A**) and 72 h (**B**) with agrimoniin at concentrations from 10^−10^ to 10^−3^ M. The viability of cells was assessed by the MTT test and presented in % of reference values. The data given represent the average values from five experiments.

Agrimoniin induced a concentration-dependent cytotoxic effect on these cancer cell lines (Figure 7). With 48-h incubation, agrimoniin IC_50_ were 1.4× 10^−6^ M and 1.29 × 10^−5^ M for K562 and HeLa cells, respectively (Figure 7A). With 72-h incubation, agrimoniin IC_50_ were 9.1 × 10^−7^ M and 9.6 × 10^−5^ M for K562 and HeLa cells, respectively (Figure 7B). In fibroblasts, no pronounced cytotoxic effect was observed over the entire concentration range of agrimoniin; maximum 20% inhibition of cell viability was reached at about 1 mM (Figure 7).

## 4. Discussion

Our studies have revealed new properties of the polyphenol agrimoniin, indicating its direct action on mitochondria. These properties include sensitization to the opening of MPTP, induction of low-amplitude swelling, activation of respiration, and reduction of an electron acceptor. The ability of agrimoniin to chelate free iron ions was also revealed, which can be essential for maintaining mitochondrial functions in different pathologies associated with iron overload. In addition to these effects on isolated mitochondria, agrimoniin manifested a cytostatic effect on K562 and HeLa tumor cells. These data are in good agreement with the mitochondria-dependent antitumor effect of agrimoniin shown earlier in cancer cell cultures [17,28]. Besides, according to our data, agrimoniin acts on cells and isolated mitochondria at comparable concentrations (about 10 μM), suggesting that mitochondria are the target of its antitumor activity.

MPTP opening is a necessary prerequisite for the activation of mitochondria-dependent apoptosis. The opening of MPTP is a mitochondrial response to calcium overloading and other cellular stresses such as oxidative stress, excess inorganic phosphate, and depletion of adenine nucleotides. As follows from our data, agrimoniin several times decreased the threshold concentrations of calcium ions inducing MPTP opening, which was accompanied by a drop in membrane potential, the release of calcium ions, and the swelling of mitochondria. It is important to note that agrimoniin itself, even at low concentrations, induced the low-amplitude swelling of mitochondria in the absence of added calcium ions. The rate and the amplitude of swelling induced by agrimoniin depended on its concentration. The swelling was entirely removed by cyclosporine A, a specific MPTP inhibitor, and partially inhibited by BHT, a lipid radical scavenger. However, a more interesting result is that the agrimoniin-induced swelling was prevented by ADP, another specific inhibitor of MPTP. This finding indicates the participation of adenylate translocase (ANT) as a key component of MPTP in the agrimoniin-induced MPTP opening. It is well-established that ADP fixes ANT in the m-state and significantly inhibits pore opening since the c-state of ANT is one of the necessary conditions for MPTP opening [38]. Consequently, the interaction with specific binding sites of ANT can underlie the influence of different agents on MPTP. It can be assumed that the effect of agrimoniin on adenylate translocase is mediated by the binding of its hydroxyl groups to individual amino acid residues of the transporter, as has been shown for other proteins and tannins [4,5,6,7,8]. This assumption is supported by our data showing that, despite the high antioxidant activity inherent in this group of compounds, agrimoniin induces swelling in the absence of other activating factors, such as an excess of calcium, reactive oxygen species, and various oxidants, which fix ANT in the c-state and stimulate MPTP opening.

An important property of agrimoniin is the ability to reduce the oxidized form of the electron acceptor DCPIP. The reduction of DCPIP is observed not only in the chemical reaction during the interaction of agrimoniin with DCPIP but also in mitochondria in the absence of an oxidation substrate. These data show that agrimoniin can be an electron donor in redox-dependent reactions in mitochondria. This property may explain the activation of respiration by agrimoniin. The activation of respiration is more pronounced during the oxidation of succinate than during the oxidation of NAD-dependent substrates. Based on these data, it can be assumed that ubiquinone, the reduction of which is associated with the oxidation of succinate, a substrate of the II complex of the respiratory chain, can be a target of the reducing effect of agrimoniin on the respiratory chain of mitochondria. Several differences in the action on the oxidative phosphorylation upon the oxidation of succinate and NAD-dependent substrates were also observed with flavonols [22]. As follows from our data, the activation of respiration is accompanied by a decrease in the efficiency of oxidative phosphorylation and, consequently, a decrease in energy production.

A valuable property of agrimoniin is the ability to chelate iron ions. Recently, this aspect has been significantly actualized in connection with identifying the mitochondria-dependent ferroptosis as a new pathway of cell death [39]. A characteristic feature of ferroptosis is iron-dependent lipid peroxidation, which mainly occurs in mitochondrial membranes [39,40]. Excessive free iron is commonly eliminated by iron chelators. Iron-chelation therapy is used to treat iron-overload disease and cancer, as well as neurodegenerative and chronic inflammatory diseases [41,42,43,44]. Although the interaction of polyphenols with iron ions is well-understood and even serves as a test for the presence of various phenols in plant extracts, including our extraction procedure, there are no data on the role of agrimoniin in iron chelation in cells or mitochondria. Our results show that agrimoniin binds iron ions with the appearance of a blue color characteristic of iron complexes with galloyl groups [45,46]. It is assumed that the formation of a complex with iron reduces the bioavailability of polyphenols for their potential targets [45], which is in good agreement with our data on the decrease in the effect of agrimoniin on the swelling of mitochondria in the presence of iron ions. These properties of agrimoniin, similar to other polyphenols, can not only provide a therapeutic basis for the chelation of excess free iron but also restrict processes of tumor growth, inflammation, and bacterial infections, which all require iron [47].

The ability of agrimoniin to activate MPTP opening and mitochondrial swelling can be realized in its cytostatic effect on tumor cells. Indeed, experiments with cancer cells have confirmed the cytotoxic effect of the isolated agrimoniin, which makes it possible to link the mitochondrial effects and the cytotoxicity. According to available data, agrimoniin IC_50_ ranges from a few μM to hundreds of μM, depending on the type of cancer cell lines. So, in mouse mammary carcinoma MM2 cells, IC_50_ was 1.36 μM and 33μM after 48-h incubation in the absence and in the presence of fetal calf serum [23]. In human gastric cancer cell SGC-7901, IC_50_ was 30 μM after 24 h incubation [17], and in human pancreatic cancer cell lines PANC-1 and CFPAC-1, IC_50_ were 269.4 and 296.8 μM, respectively, after 24-h incubation [48]. In our study, we tested hormone-dependent tumor cells K562 and HeLa. In these tumors, the activation of mitochondria-dependent apoptosis can be critical in the induction of cell death. As follows from our data, agrimoniin induced a concentration-dependent cytotoxic effect on K562 and HeLa cells and only weakly affected fibroblasts. IC_50_ values for these cancer cells were in line with the above literature data, being in the range 1.4–96 μM. The low cytotoxicity towards skin fibroblasts agrees with the previously published data showing that agrimoniin-containing strawberry extract pretreatment increased human dermal fibroblast viability [49].

Since the activating influence of agrimoniin on MPTP opening is removed by ADP, it can be assumed that ANT is involved in this process. This assumption explains the observed differences in the action of agrimoniin on tumor cells and fibroblasts since it is known that the overexpression or the knockdown of ANT isoforms modulates the sensitivity of cells to apoptotic stimuli. In this context, it is also important to emphasize that different isoforms have opposite effects on cell survival [38,50]. The proapoptotic isoform ANT1 was found to be low in many cancers, while the induction of overexpression of ANT1 in breast cancer promoted tumor apoptosis [51]. ANT1 and ANT3 isoforms act as proapoptotic factors, while ANT2 and 4 isoforms provide the resistance to death-inducing stimuli [52,53]. ANT2 is either absent or weakly expressed in all tissues, while ANT3 is expressed in tissues and cultured fibroblasts in the amount that depends on the metabolic status [54]. Thus, differences in the expression of specific ANT isoforms can determine the sensitivity of the tested cell lines to agrimoniin-induced MPTP opening, which requires further study.

Several pathways of anticancer activity of agrimoniin and other hydrolyzable tannins have been identified at different times. Some of these apoptotic pathways are associated with a decrease in the expression of the antiapoptotic protein Bcl-2 and an increase in the expression of pro-apoptotic caspase-3, Bax, P53, and cytochrome C proteins, as was shown on porcine intestinal IPEC-J2 cells and bladder cancer T24 cells [18,19]. These disturbances in gene expression were accompanied by the suppression of PI3K/Akt/NF-κB signaling pathways. As was shown on human gastric cancer SGC-7901 cells, agrimoniin induced the cytosolic calcium imbalance, increased the intracellular level of reactive oxygen species (ROS), decreased the mitochondrial transmembrane potential, and promoted apoptosis [17]. The new pathway of agrimoniin cytotoxicity determined by metabolism dysfunction was recently identified on pancreatic cancer cells [48]. It was found that agrimoniin induced an increase in the production of ROS through the suppression of the Nrf2-dependent ROS scavenging system, which led to a substantial decline in energy metabolism, including both glycolysis and oxidative phosphorylation. Thus, after 24-h incubation with these cells, agrimoniin at concentrations of 100–300 µM almost completely suppressed respiration, glycolysis, and ATP production. Interestingly, all of the above effects were prevented after the pretreatment of cells with the thiol antioxidant N-acetylcysteine, as was noted for both pancreatic cancer cells and bladder cancer T24 cells [19,48]. Based on these data, it can be assumed that the cytotoxic effect of agrimoniin on the cancer cells may depend, in particular, on the redox state of cells and mitochondria. This assumption agrees with our data showing that one of the most interesting properties of agrimoniin is the ability to reduce electron acceptors.

**Scheme 1:**
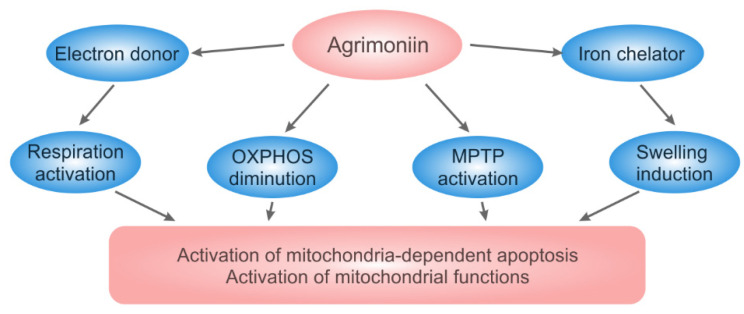
The properties and mitochondrial effects of agrimoniin. Agrimoniin supplies electrons to the respiratory chain, stimulates mitochondria-dependent apoptosis via activation of the swelling and MPTP opening, and chelates iron ions.

Scheme 1 summarizes the properties and mitochondrial effects of agrimoniin, which have been identified in our study. It is important to emphasize that all these effects manifest themselves convincingly even at low concentrations of agrimoniin. This influence on mitochondria can stimulate apoptosis of cancer cells or activate mitochondrial functions of normal cells. Due to these properties, agrimoniin can also be helpful in the prevention and removal of stagnant pathological states.

## 5. Conclusions

The highly active polyphenol agrimoniin was obtained after multistage purification of the extract from *A. pilosa* Ledeb. The study has revealed new properties of agrimoniin associated with its direct action on mitochondria, including the sensitization to the opening of MPTP, induction of low-amplitude swelling, and activation of respiration with a decrease in energy production. All these properties can provide the activation of mitochondria-dependent apoptosis or increase the sensitivity to different factors stimulating apoptosis, in particular, to calcium overload. The data suggest that mitochondria are a target of the antitumor activity of agrimoniin, observed in our and other studies. It is possible that the effects of agrimoniin on mitochondria are based on the interaction of its numerous hydroxyl groups with adenylate translocase, a key regulator of both MPTP and oxidative phosphorylation. In this case, the activation of mitochondria-dependent apoptosis will depend on the expression of adenylate translocase isoform specific for a distinct cancer cell line.

A valuable property of agrimoniin is the ability to bind free iron since the chelation can restrict tumor growth, inflammation, and bacterial infections, which all require iron. The ability to reduce electronic acceptors indicates the possibility of the activation of respiration at the level of the complex II of the respiratory chain and the shunting of the first complex, which requires additional research.

## Abbreviations

Ag	agrimoniin
ANT	adenylate translocase
BHT	butylhydroxytoluene
CRC	calcium retention capacity
Cs A	cyclosporin A
DNP	2,4-Dinitrophenol
DCPIP	2,6-Dichlorophenolindophenol
MPTP	mitochondrial permeability transition pore
MTT	3-(4,5-Dimethylthiazol-2-yl)-2,5-diphenytetrazolium bromide
PMS	phenazinemethosulfate
SDH	succinate dehydrogenase
TPP+	tetraphenylphosphonium
